# Automatic virtual reconstruction of acetabular fractures using a statistical shape model

**DOI:** 10.1007/s00068-024-02615-7

**Published:** 2024-08-27

**Authors:** WA van Veldhuizen, R van Noortwijk, AML Meesters, K ten Duis, RCL Schuurmann, JPPM de Vries, JM Wolterink, FFA IJpma

**Affiliations:** 1 Department of Surgery, University Medical Centre Groningen, Groningen, The Netherlands; 2 3D lab, University of Groningen, University Medical Center Groningen, Groningen, The Netherlands; 3 Multimodality Medical Imaging Group, Technical Medical Center, University of Twente, Enschede, The Netherlands; 4 Department of Applied Mathematics, Technical Medical Center, University of Twente, Enschede, The Netherlands

**Keywords:** Acetabular fracture, Statistical shape model, Virtual reconstruction, Contralateral mirroring

## Abstract

**Purpose:**

Automatic virtual reconstruction of complex fractures would be helpful for pre-operative surgical planning. We developed a statistical shape model (SSM) which contains data of 200 intact 3D hemipelves. It allows for quantification of shape differences and is able to reconstruct abnormal shaped pelvises. We applied our SSM to reconstruct elementary and associate type acetabular fractures and assessed the reconstruction performance of the SSM, by comparing the reconstructed shape with the intact contralateral hemipelvis.

**Methods:**

In this retrospective diagnostic imaging study, we used our SSM to virtually reconstruct fractured hemipelves of eighty-three patients with an acetabular fracture. A root mean square error (RMSE) was computed between the reconstructed shape and intact contralateral shape for the whole hemipelvis and for regions relevant for plate-fitting. These plate-fitting relevant regions were defined as: (1) Iliopectineal line length and radius; (2) ischial body line length and radius; (3) acetabular diameter, (4) quadrilateral slope and (5) weight-bearing acetabular dome.

**Results:**

The median RMSE of the whole hemipelvis of the elementary type fractures was 2.2 (1.7–2.5) mm versus 3.2 (2.2–3.9) mm for the associate type fractures (*p* < 0.001). The median RMSE for the plate-fitting regions of elementary type fractures was 1.7 (1.4–2.1) mm versus 2.7 (2.0–4.1) mm for associate type fractures (*p* < 0.001).

**Conclusion:**

Using a statistical shape model allows for accurate virtual reconstructions of elementary and associate type acetabular fractures within a clinically acceptable range, especially within regions important for plate-fitting. SSM-based reconstructions can serve as a valuable tool for pre-operative planning in clinical practice, when a template of the contralateral hemipelvis is unavailable.

**Supplementary Information:**

The online version contains supplementary material available at 10.1007/s00068-024-02615-7.

## Introduction

Acetabular fractures occur in young individuals due to high-energy trauma and in the frail elderly due to low-energy trauma combined with osteoporotic bone [1]. Acetabular fractures are considered complex fractures, due to their three-dimensional (3D) geometry and the displacement of fracture fragments in different directions [2]. Open reduction and internal fixation (ORIF) is the standard treatment for displaced acetabular fractures, with the aim of avoiding post-traumatic arthritis and preserving the long-term functionality of the hip joint. According to Verbeek et al. (2018) a successful post-operative outcome is achieved if the desired gap value is within a limit of 5 mm and the step-off value is within a limit of 1 mm, to avoid conversion to total hip arthroplasty (THA) [3]. The gap value was defined as the separation of fracture fragments along the articular surface, whereas step-off is defined as this separation perpendicular to the circumference of the acetabular dome [4].

Pre-operative virtual surgical planning of acetabular fractures is increasingly used in clinical practice since it is expected to reduce operation time and to provide more accurate reconstructions [5]. By using a 3D printed template obtained from a computed tomography (CT)-based virtual fracture model, a reconstruction plate can be pre-contoured to fit the patient-specific anatomy, thereby simplifying the surgical procedure [6]. Virtual reconstruction can be performed by mirroring the intact contralateral hemipelvis, serving as a template for pre-contouring [7]. However, in cases of bilateral acetabular fractures, combined acetabular and pelvic ring injuries (i.e. occurring in up to 16% of cases with pelvic injuries), in-situ implants (e.g. screws, nails, plates) at the contralateral side, or a highly asymmetric contralateral hemipelvis, the contralateral hemipelvis cannot be used as a template for virtual fracture reduction [8]. Based on our clinical experience, this happens on a weekly basis in our level 1 trauma centre. In these cases, virtual reconstruction of the pelvis by means of a statistical shape model (SSM) can be used, as was proposed by De Angelis et al. (2023) and Krishna et al. (2022) [9, 10]. These studies were, however, based on small datasets or involved only computer-simulated fractures. Therefore, there is a need to explore the use of SSMs for the reconstruction of complex acetabular fractures observed in clinical practice within a more extensive patient cohort.

The main objectives of this research were to use an SSM to reconstruct elementary and associate type acetabular fractures and to compare this reconstructed shape with its intact contralateral hemipelvis by assessing the reconstruction performance of both the whole hemipelvis and the regions of interest for plate-fitting.

## Materials and methods

This retrospective diagnostic imaging study was reviewed, and a waiver was provided by the Medical Ethics Review Committee of the University Medical Center Groningen, no: 2016.385. This study is reported following Strengthening the Reporting of Observational Studies in Epidemiology (STROBE) guidelines and was performed in line with the Declaration of Helsinki [11].

### Study population

A total of eighty-three patients, who were treated between 2005 and 2020 for a unilateral acetabular fracture in a level 1 trauma centre, were included. These patients had all types of acetabular fractures representing clinical practice, including both elementary as well as associate types. Patients were included based on the availability of a pre-operative CT-scan with a maximum slice thickness of 2 mm. Patients treated with a primary THA, under 18 years old, with a periprosthetic fracture, or patients who had a concomitant pelvic ring injury, a pipkin femoral head fracture, or insufficient segmentations of the pelvis were not eligible for this study.

### Preprocessing of segmentations

The imaging data was retrieved from a previous study by Meesters et al. (2022) [12]. Baseline characteristics were retrieved from the patients’ medical records. Pre-operative CT data was used to obtain segmentations of both the fractured and contralateral hemipelves, using segmentation-certified software of Mimics Medical (version 19.0; Materialise, Leuven, Belgium). All fracture fragments were segmented using a pre-set threshold for bone (≥ 226 Hounsfield Units) and were saved in separate segmentations. To ensure a closed surface of each segmentation, a wrapping operation of each fragment was performed in the 3D software 3-Matic (version 17.0; Materialise, Leuven, Belgium). For each patient, the different wrapped fracture fragments were saved into one segmentation.

To perform principal component analysis (PCA), all segmentations were aligned and registered to enable point-to-point correspondence. Both the fractured and intact contralateral shapes were remeshed with an isotropic edge length of 1.5 mm using a remesher function implemented in Matlab (MATLAB 2023a, The MathWorks, Inc., MA, USA) [13]. This is to ensure a uniform distribution of coordinates in each segmentation, which is a requirement for principal component analysis. Accordingly, a non-rigid iterative closest point (ICP) algorithm was used to register each segmentation to a template segmentation, as was described previously by our study group [14].

### Statistical shape modelling

**Fig. 1:**
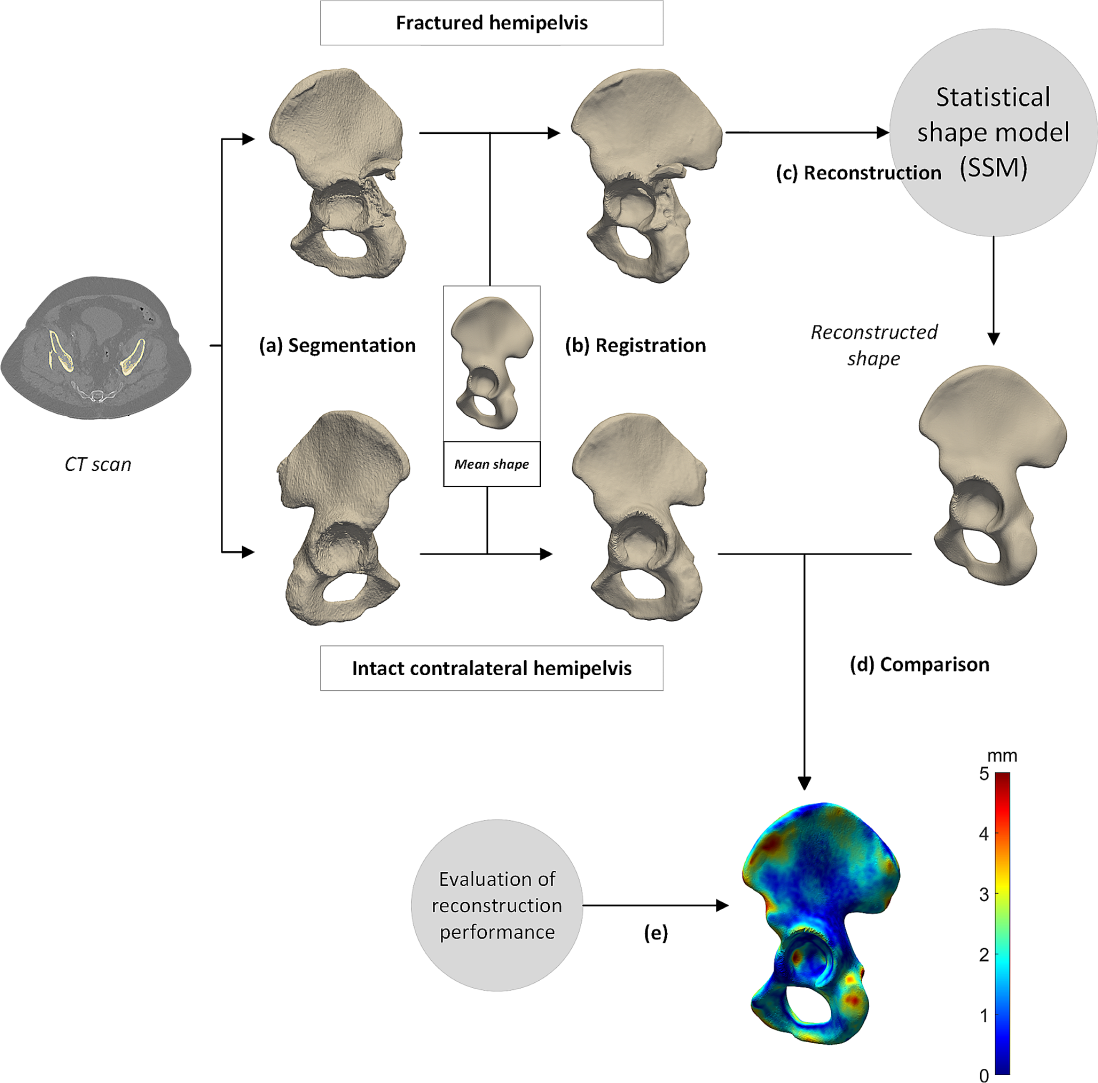
Workflow of the reconstruction process. A computed tomography (CT-)scan (**a**) was used to obtain two segmentations, one of the fractured hemipelvis and one of the intact contralateral hemipelvis (**b**). Both the fractured hemipelvis and the intact contralateral hemipelvis were registered to the mean template shape, this process includes smoothing and mirroring. (**c**) The registered segmentation of the fractured hemipelvis was reconstructed by the statistical shape model (SSM), so a reconstructed shape was obtained from the SSM. (**d**) This reconstructed shape was compared to the registered intact contralateral hemipelvis and a distance map was generated. The distance map displays the difference between the two shapes by colour (blue represents a small difference, orange a large difference) and (**e**) the reconstruction performance was assessed for the whole hemipelvis and for the regions relevant for plate-fitting

The SSM, previously developed by our research group, is based on 200 intact left hemipelves and consists of 100 female patients and 100 male patients [14]. The distributions of age, height and weight were comparable to the distributions in the Dutch population [15, 16]. This SSM, consisting of the first fifteen principal components (PCs), was used to reconstruct a fractured hemipelvis. To reconstruct the fractured hemipelvis, the mean shape was used as a template for the SSM. For registration purposes, this same mean shape was used to register the contralateral hemipelvis, ensuring the same number of coordinates for the reconstructed and contralateral shapes. Figure 1 describes the workflow from a CT scan to reconstruction of the fractured hemipelvis by the SSM.

### Evaluation of reconstruction performance

**Fig. 2:**
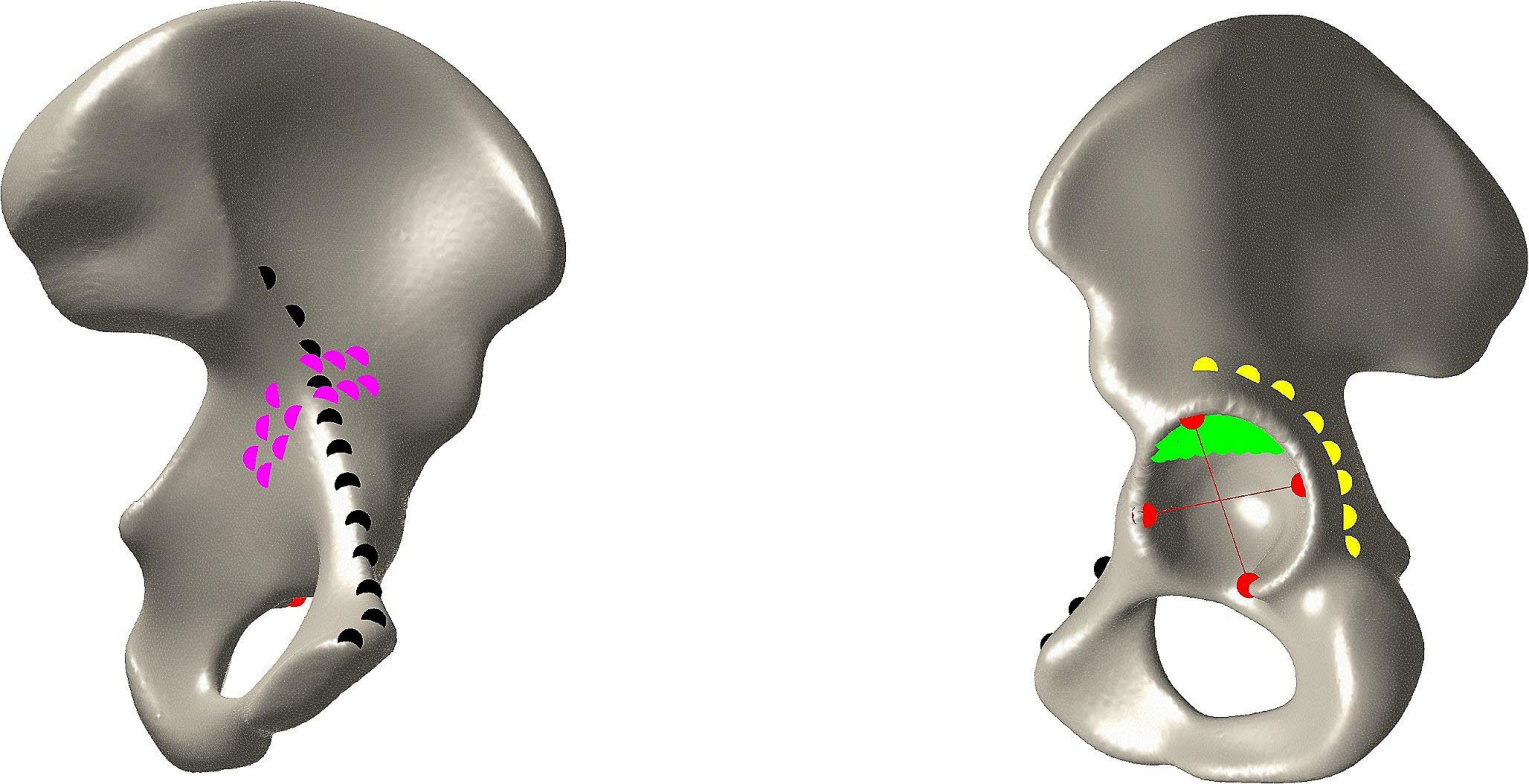
Manually placed coordinates (*n* = 66) to evaluate clinically relevant regions regarding plate-fitting and pre-operative virtual planning. (**a**) Iliopectineal line coordinates (black dots, *n* = 12) and quadrilateral slope (pink dots, *n* = 12). (**b**) Ischial body line coordinates (yellow dots, *n* = 8), acetabular diameter (red dots, *n* = 4) and representation of the weight-bearing acetabular dome (green dots, *n* = 30). The coordinates are projected on the mean shape

A root mean square error (RMSE) was calculated between the reconstructed shape and the intact contralateral shape, for the hemipelvis as a whole and for the coordinates (*n* = 66) within the regions relevant for plate-fitting (Fig. 2). The distance map in Fig. 1(e) shows the colour-coded difference in terms of RMSE of the whole hemipelvis.

### Quantification of clinically relevant regions

Clinically relevant regions regarding plate-fitting were defined by an experienced trauma surgeon (FIJ) to assess the reconstruction performance of the SSM. The following parameters were defined: (1) Iliopectineal line length and radius; (2) ischial body line length and radius; (3) acetabular diameter, (4) quadrilateral slope (Fig. 2). In these regions, both the difference in length, radius, diameter and slope, as well as the RMSE, were assessed. Furthermore, the RMSE was calculated for the coordinates representing the weight-bearing acetabular dome, presented as the green dots in Fig. 2.

Initially, after manual placement of coordinates onto the general mean shape, corresponding to the clinically relevant regions for positioning of implants, the indices of these coordinates were saved. Consequently, these indices were used to obtain the coordinates of each reconstructed shape and its corresponding intact contralateral shape. By means of this, the parameters could be automatically calculated for both shapes. Computation of these parameters are described in further detail in Online Resource 1.

### Statistical analyses

Data are presented as median with interquartile range (IQR; 25th and 75th percentile). Differences between the elementary and associate type fractures were tested with a Mann-Whitney U test. The primary endpoints of the study were the computation of the RMSE for the whole hemipelvis and the RMSE of the regions relevant for plate-fitting. The secondary endpoint was the computation of the six parameters defined in the plate-fitting regions. Statistical analyses were performed using Python (version 3.11), with *p*-values < 0.05 considered significant.

## Results

### Study population

A total of eighty-three patients with a median age of 49 (38–63) years were included. Most of them sustained a both column acetabular fracture representing one of the most complex acetabular fracture patterns (Table 1).

**Table 1:** Distribution of fracture types

**Elementary type fractures (no.)**	
Anterior column	6
Posterior column	1
Posterior wall	17
**Associated type fractures (no.)**	
Anterior column and posterior hemitransverse	5
Both column	28
Posterior column and posterior wall	7
T-type	7
Transverse and posterior wall	12
**Total**	**83**

### Evaluation of reconstruction performance

Table 2 shows the reconstruction performance in terms of RMSE between the reconstructed shape and its corresponding intact contralateral shape, evaluated on the whole hemipelvis and the plate-fitting regions. The median RMSE of the whole hemipelvis of the elementary type fractures was 2.2 (1.7–2.5) mm versus 3.2 (2.2–3.9) mm for the associate type fractures (*p* < 0.001). The median RMSE for the plate-fitting regions of elementary type fractures was 1.7 (1.4–2.1) mm versus 2.7 (2.0–4.1) mm for associate type fractures (*p* < 0.001).

**Fig. 3:**
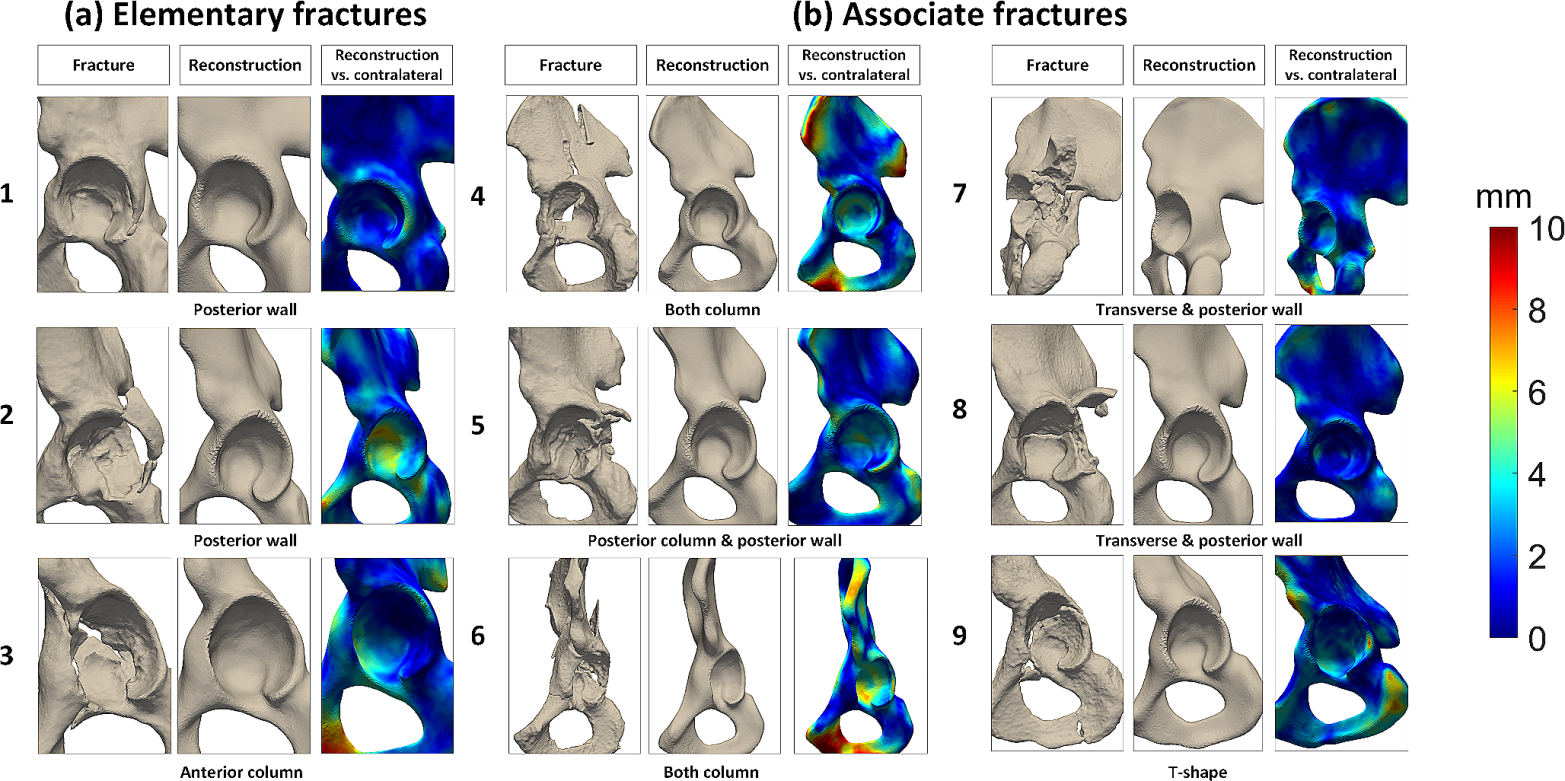
Visualization of the reconstruction performance of the SSM for (**a**) three elementary (cases 1–3) and (**b**) six associate (cases 4–9) type fractures. The first plot shows the fractured shape and the second plot the reconstructed shape. The third plot shows the difference between the reconstructed and contralateral shapes as a distance map, with blue representing small differences (0–2 mm) and red representing large differences (10 mm). Larger deviations are found in ilium and pubic regions, e.g. shown in cases 3, 4, 6 and 9

Figure 3 visualizes the reconstruction of the SSM from a fractured hemipelvis and provides a surface distance by means of a distance map for different elementary and associate type fractures. Larger deviations were found in ilium and pubic regions, which can also be observed in cases 3, 4, 6 and 9 (Fig. 3).

**Table 2:** Reconstruction performance (root mean square error; RMSE) of the statistical shape model compared to the intact contralateral hemipelvis, for the whole hemipelvis and the plate-fitting regions

Fracture type	No. of patients (%)	RMSE (mm)– whole hemipelvis*	RMSE (mm)– plate-fitting regions*
*Elementary type fractures*			
Anterior column	6	2.8 (2.3–3.2)	2.6 (2.3–2.9)
Posterior column	1	1.7	1.6
Posterior wall	17	2.1 (1.7–2.3)	1.5 (1.2–1.8)
***Total elementary***	***24 (29)***	***2.2 (1.7–2.5)***	***1.7 (1.4–2.1)***
*Associate type fractures*			
Anterior column + posterior hemitransverse	5	3.2 (2.5–3.5)	2.6 (2.6–3.6)
Both column	28	3.8 (3.4–4.9)	4.1 (3.3–5.3)
Posterior column + posterior wall	7	2.0 (2.0–2.7)	1.9 (1.6–2.0)
T-type	7	2.7 (2.2–2.9)	2.5 (1.9–3.6)
Transverse + posterior wall	12	2.4 (1.9–3.2)	2.4 (1.5–2.6)
***Total associate***	***59 (71)***	***3.2 (2.2–3.9)***	***2.7 (2.0–4.1)***
**Total**	**83**	**2.7 (2.1–3.6)**	**2.4 (1.7–3.6)**

Values are represented as no. (%) or median (IQR; 25th and 75th percentile). RMSE = root mean square error

*Each value represents the difference between the reconstructed shape and its corresponding intact contralateral shape

### Evaluation of parameters within clinically relevant regions for plate-fitting

Table 3 shows the results of the analysis based on parameters of the clinically relevant regions for plate-fitting, for elementary and associate type fractures separately.

**Table 3:** Differences between the reconstructed shape and the contralateral shape of elementary and associate type fractures. The plate-fitting parameters include iliopectineal line length and radius, ischial body line length and radius, acetabular diameter, and quadrilateral slope

	Elementary type fractures (*n* = 24)	Associate type fractures (*n* = 59)
Iliopectineal line length (mm)	2.6 (1.2–3.5)	4.0 (1.5–6.1)
Iliopectineal line radius (mm)	9.7 (6.2–15.8)	38.5 (16.5–64.5)
Ischial body line length (mm)	1.9 (1.1–2.6)	1.9 (0.9–4.0)
Ischial body line radius (mm)	2.1 (1.1–2.7)	2.0 (0.9–4.7)
Acetabular diameter (mm)	1.0 (0.6–1.5)	1.8 (0.9–3.0)
Quadrilateral slope (⁰)	6.3 (3.8–8.3)	6.6 (2.9–10.4)

Values are represented as median (IQR; 25th and 75th percentile)

### Case studies illustrating clinical use

**Fig. 4:**
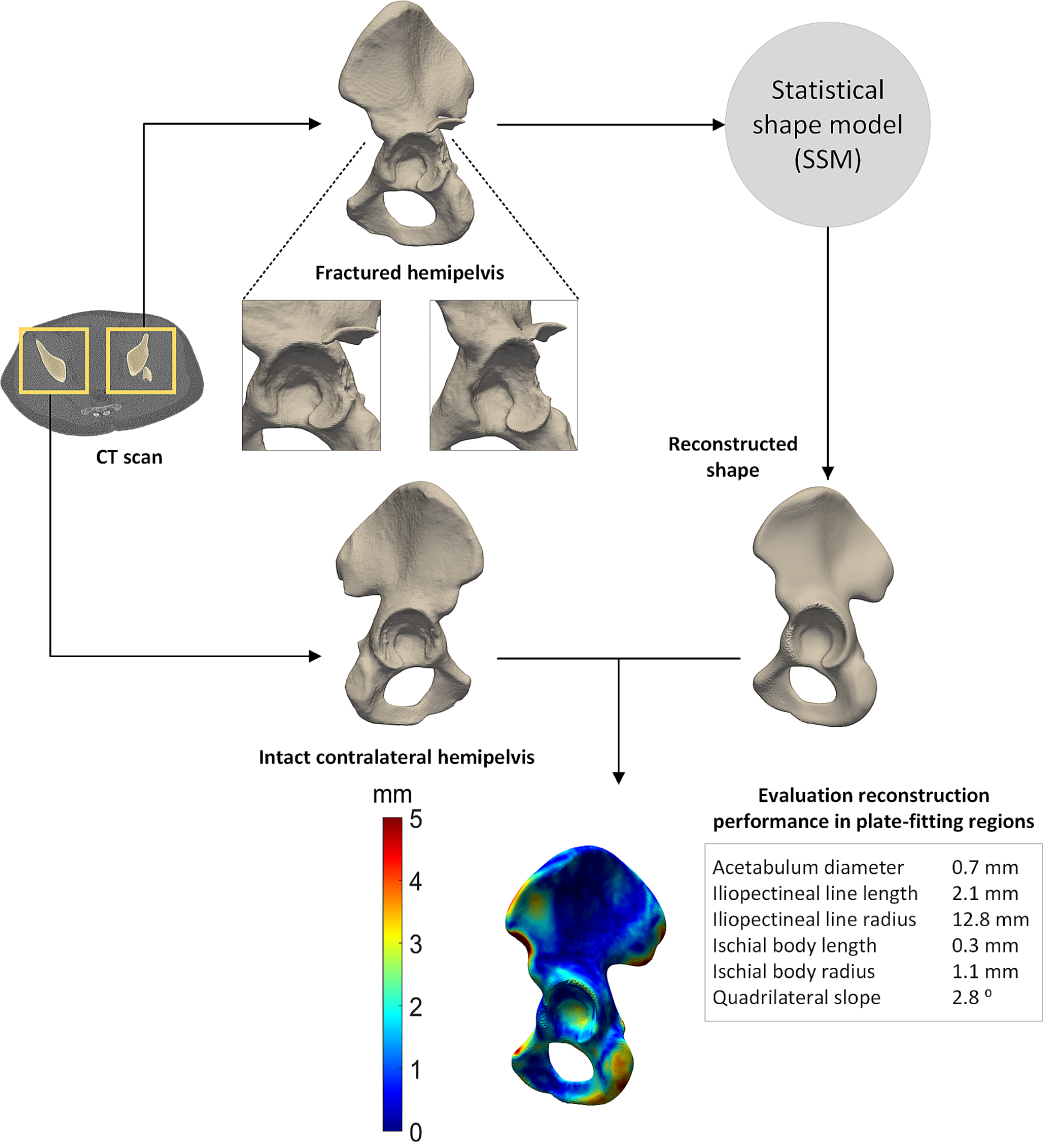
Clinical case illustrating a statistical shape model (SSM)-based reconstruction of an elementary type acetabular fracture. Fractured shape and intact contralateral shapes were segmented from the computed tomography (CT) scan. For clarity purposes the registration step, as was described in Fig. 2, was omitted in the current figure. The SSM provided a reconstructed shape from the fractured hemipelvis. Accordingly, the reconstruction performance was evaluated, by comparing the intact contralateral shape with the reconstructed shape. The distance map shows deviations between these two shapes, with blue representing small differences (0–2 mm) and red representing large differences (5 mm). The results of the parameters representing the plate-fitting regions are displayed in the grey box. Each value represents the difference between the reconstructed and intact contralateral shape

The first case study describes the reconstruction of a posterior wall acetabular fracture in a 21-year-old male patient. The patient had a car accident, which ended in a collision with a tree, and he suffered from an acetabular fracture. Figure 4 shows how the SSM can be used in clinical practice, by using the preoperative CT scan. The fractured hemipelvis is reconstructed by the SSM, resulting in an accurate representation of how this hemipelvis would have looked like before the fracture. The total mean ± standard deviation RMSE of this patient was 1.4 ± 1.0 mm, whereas the mean RMSE of the plate-fitting relevant regions was 1.0 ± 0.5 mm. These values indicate a very accurate reconstruction performance of the displaced posterior wall fracture by the SSM.

**Fig. 5:**
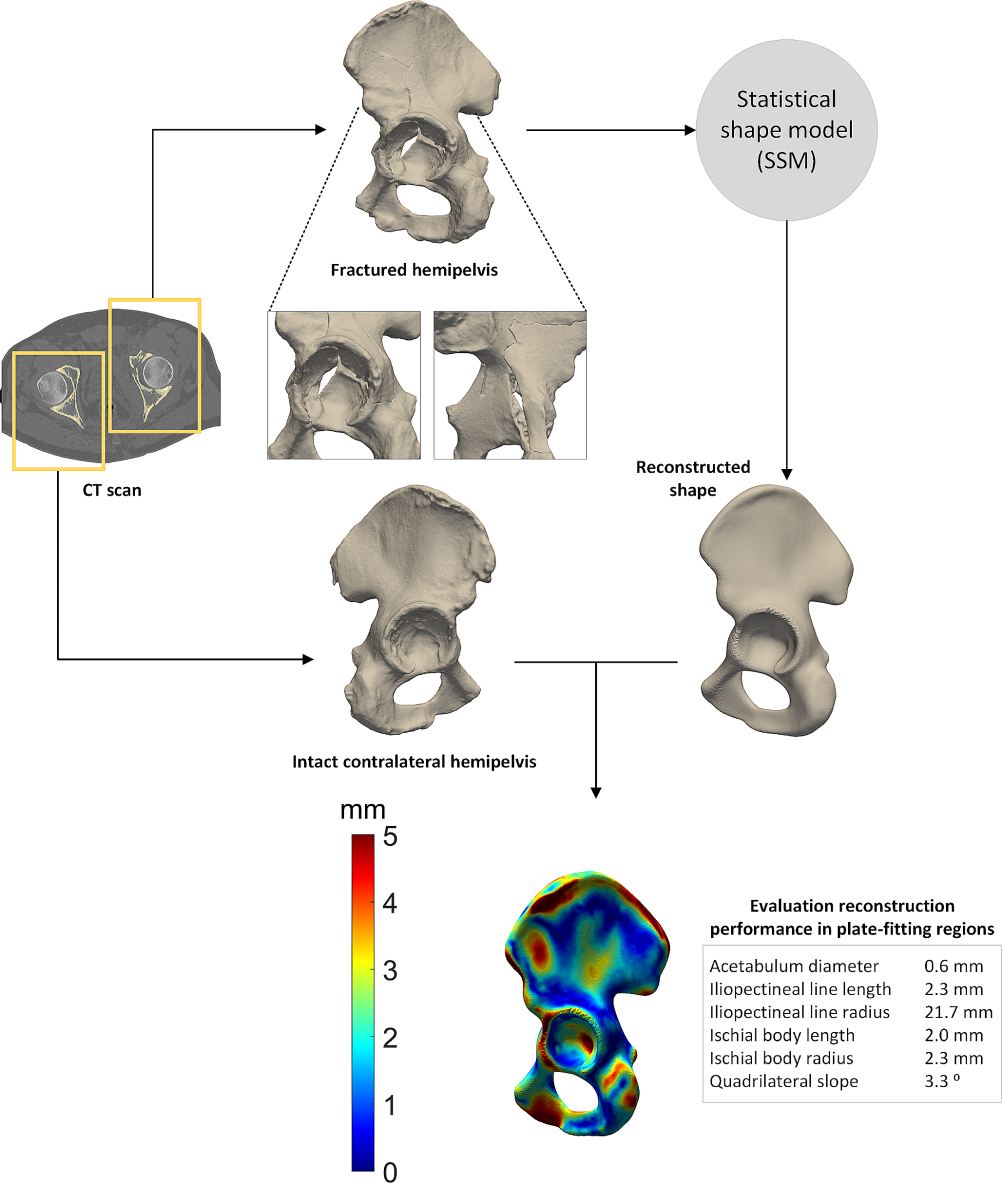
Clinical case illustrating a statistical shape model (SSM)-based reconstruction of an associate type acetabular fracture. Fractured shape and intact contralateral shapes were segmented from the computed tomography (CT) scan. For clarity purposes the registration step, as was described in Fig. 2, was omitted in the current figure. The SSM provided a reconstructed shape from the fractured hemipelvis. Accordingly, the reconstruction performance was evaluated, by comparing the intact contralateral shape with the reconstructed shape. The distance map shows deviations between these two shapes, with blue representing small differences (0–2 mm) and red representing large differences (5 mm). The results of the parameters representing the plate-fitting regions are displayed in the grey box. Each value represents the difference between the reconstructed and intact contralateral shape

The second case study describes the reconstruction of a both column acetabular fracture in a 48-year-old male patient. The patient suffered from a displaced acetabular fracture following a fall from his race bicycle. The patient was treated with open reposition by means of the anterior pelvic approach. Fracture reduction and fixation were done with a suprapectineal plate, without postoperative complications. Figure 5 shows how the SSM can be used in clinical practice, by using the preoperative CT scan. The fractured hemipelvis is reconstructed by the SSM, resulting in an accurate representation of how this hemipelvis would have looked like before the fracture. The total mean ± standard deviation RMSE of this patient was 2.2 ± 1.4 mm, whereas the mean RMSE of the plate-fitting relevant regions was 1.7 ± 1.0 mm. These values indicate an accurate reconstruction performance of the displaced both column fracture by the SSM.

**Fig. 6:**
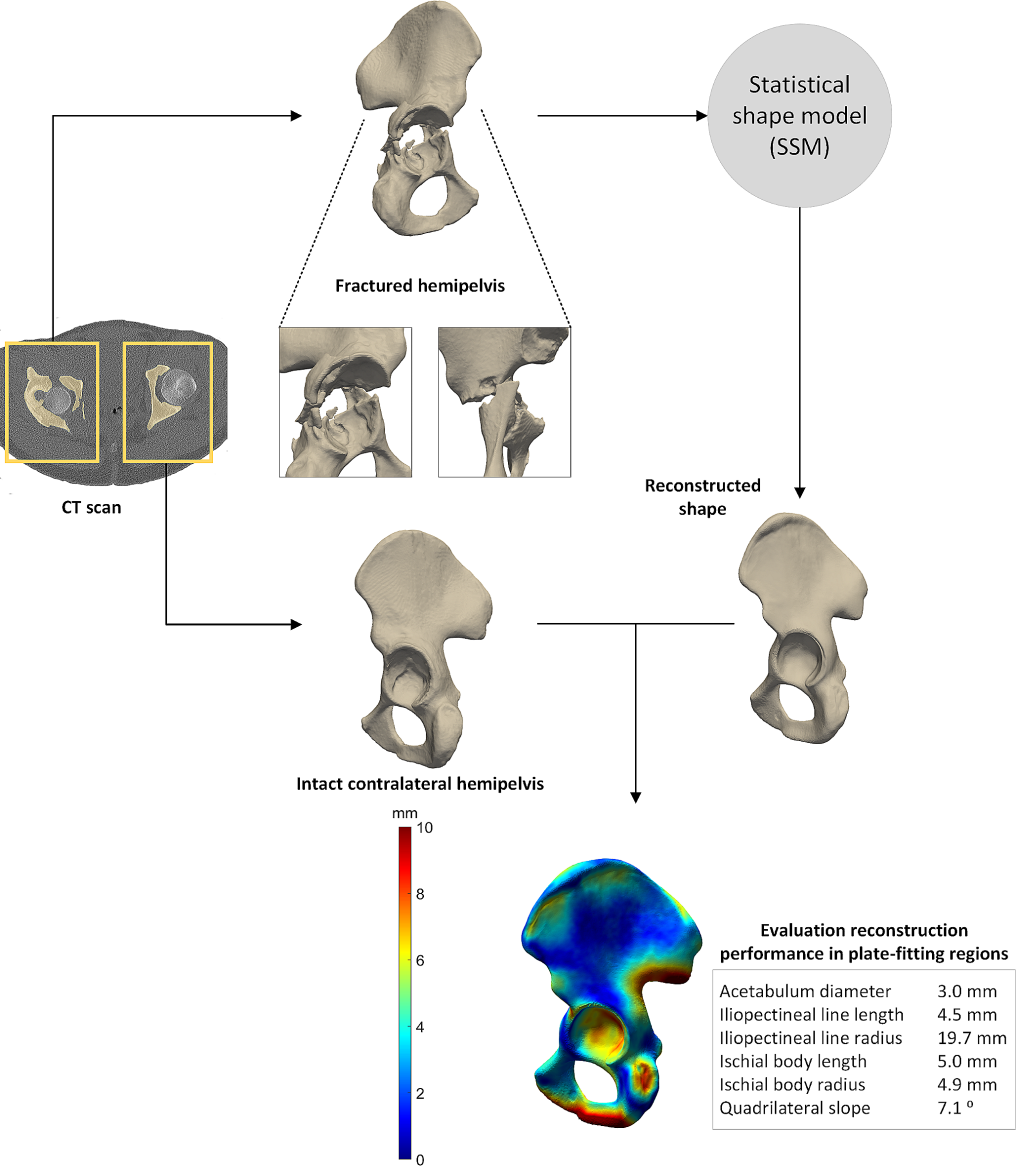
Clinical case illustrating a statistical shape model (SSM)-based reconstruction of an associate type acetabular fracture. Fractured shape and intact contralateral shapes were segmented from the computed tomography (CT) scan. For clarity purposes the registration step, as was described in Fig. 2, was omitted in the current figure. The SSM provided a reconstructed shape from the fractured hemipelvis. Accordingly, the reconstruction performance was evaluated, by comparing the intact contralateral shape with the reconstructed shape. The distance map shows deviations between these two shapes, with blue representing small differences (0–3 mm) and red representing large differences (10 mm). The results of the parameters representing the plate-fitting regions are displayed in the grey box. Each value represents the difference between the reconstructed and intact contralateral shape

The third case study describes the reconstruction of a transverse and posterior wall fracture in a 25-year-old male patient. The patient suffered from a displaced acetabular fracture due to a collision with a stationary car. The patient was treated operatively by means of a Kocher-Langebeck approach for reposition and fixation of the posterior column and posterior wall. Later the anterior, intra-pelvic approach was performed, without any post-operative complications. Figure 6 shows how the SSM can be used in clinical practice, by using the pre-operative CT scan. The fractured hemipelvis was reconstructed by the SSM, resulting in an accurate representation of how this hemipelvis would have looked like before the fracture. The total mean ± standard deviation RMSE of this patient was 3.7 ± 2.1 mm, whereas the mean RMSE of the plate-fitting relevant regions was 2.7 ± 1.3 mm. These values indicate a less accurate reconstruction performance of the highly displaced transverse and posterior wall fracture by the SSM.

## Discussion

The main objectives of this study were to reconstruct acetabular fractures by means of an SSM and to assess its reconstruction performance by comparing the reconstructed shape with the intact contralateral shape. Median RMSE of the whole hemipelvis as well as the plate-fitting regions were within clinically acceptable ranges for both elementary fractures and associate type fractures. A clinically acceptable gap threshold has previously been defined at 5 mm [3]. Also, the lengths and diameters of the regions relevant for plate-fitting were all within the 5 mm threshold. Only the iliopectineal line radius showed some deviation for associate type fractures, but it should be noted that larger deviations in radius values do not necessarily negatively influence clinical usability. Differences in quadrilateral slope were similar for elementary and associate type fractures, both with median values of approximately 6⁰, and were within a clinically acceptable range to adequately pre-contour a plate. The clinical relevance of being able to perform accurate SSM-based reconstructions of acetabular fractures is that in cases of bilateral fractures, or when the contralateral hemipelvis is unavailable for mirroring, the SSM can reconstruct elementary type fractures with excellent accuracy and associate type fractures with good accuracy.

Previous work on the use of an SSM for virtual reconstruction includes two studies by Ead et al. (2020 and 2021). The first study describes virtual reconstruction by using the mirrored contralateral hemipelvis as a template (*n* = 8) and the second study describes the use of an SSM to reconstruct acute unilateral fractures (*n* = 8) [5, 17]. They showed some preliminary results of SSM-based acetabular fracture reduction in 8 patients. Their reconstruction procedure, however, differed from ours, since we used the SSM to reconstruct a shape given multiple fracture fragments as input, whereas they realigned the fracture fragments to the template shape provided by their SSM. Our study adds to previous literature because we presented an automatic workflow from a segmentation of a CT-scan to a reconstructed shape for different acetabular fracture types. Moreover, we evaluated eighty-three fractures instead of case series presented in previous studies.

Krol, Skadlubowicz, Hefti & Krieg (2013) virtually reconstructed eight tumour-damaged pelvic bones with a sex-specific SSM (50 male and 50 female subjects) and compared this reconstruction performance with the contralateral mirroring method [18]. They showed that the SSM reconstructs the defect with the same clinically acceptable accuracy as the mirroring method. They provided a mean RMSE value of 1.26 ± 1.08 mm for the entire hemipelvis, whereas our research added an analysis of plate-fitting specific regions, providing a more detailed insight into differences in those regions. During development of our SSM, initially the reconstruction was performed with a sex-specific mean shape as a template. However, the median (IQR) RMSE of the reconstruction template did not differ between a general mean shape (2.3 (2.0–3.6) mm) or a sex-specific mean shape (2.2 (2.0–3.7) mm). Therefore, we decided to use the general mean shape as the template shape for the SSM-based reconstruction. Another study by Krishna et al. (2022) compared two reconstruction methods, namely contralateral mirroring and reconstruction with an SSM (consisting of 33 female hemipelves) [9]. They artificially generated three different cuts in the intact left hemipelvis, simulating different kinds of acetabular fractures. They conclude that contralateral mirroring is more accurate than SSMs for reconstructing unilateral pelvic fractures. However, their study design differed from ours, since we used contralateral mirroring as a ground truth to compare the SSM’s reconstruction performance with, instead of comparing the RMSE values of contralateral mirroring with the SSM’s reconstructed shapes. Moreover, their study was based on computer-simulated fractures, whereas our study evaluated the SSM’s reconstruction performance on real and complex clinical cases.

The difference in reconstruction performance between the elementary and associate type fractures should be attributed to more displacement of multiple fracture fragments in associate type fractures as compared to elementary type fractures. For both type of fractures, the median RMSE values of the whole hemipelvis were higher compared to the median RMSE of the plate-fitting regions, indicating that a higher reconstruction error is found in those regions less important for plate-fitting. The reconstruction performance of patients with associate type fractures differed substantially among individuals in this group, with those having large displaced fracture fragments generally showing lower reconstruction performances. In this study, all separate fracture fragments that were segmented from the CT scan were combined and used as input for the SSM. Another option would be to provide only the largest fracture fragments as input for reconstruction. However, the performance will not improve for all cases, since the SSM needs a specific proportion of the total size as input. Nolte and Bull (2019) showed that large defects, i.e. >25% of missing data, resulted in less accurate reconstructed shapes compared to the contralateral shape [19]. To improve our reconstruction performance for associate type fractures, for which generally more intact surface is missing, we could manually reposition the fragments prior to reconstruction by the SSM. However, manual repositioning takes approximately thirty minutes, and there is a need for a technical physician to perform this manual repositioning. In the future, instead of manual repositioning of the fracture fragments, a CNN-based or SSM-based pre-processing step could be implemented within the automated pipeline as presented in this study, to provide automatic repositioning of large displaced fracture fragments [20, 21]. Accordingly, medical centres without a dedicated lab to perform these tasks would still be able to obtain accurate reconstructions using an SSM.

As a future perspective, besides its use as a reconstruction tool, the SSM can also be used for other clinical purposes. One of these purposes is related to plate-fitting. Prior to surgery, 3D models can be used to pre-contour an off-the-shelf plate to fit the patient-specific anatomy, or these models can be used to develop patient-specific implants [6, 22–24]. If the contralateral hemipelvis is unavailable for these purposes, a reconstructed shape by the SSM can be used. The SSM can provide different mean shapes, related to sex-specific and/or size-specific shapes. An off-the-shelf plate can be fitted to each of these shapes, thereby evaluating the use case of fitting adequacy for different subpopulations. Because this is a relatively new development, the software to obtain SSM-based reconstructions is not yet publicly available. However, in a previous study we provided the general, female and male mean shapes as stereolithography (STL) files [14]. To clarify this concept, we provide some documentation in Online Resource 2 explaining the steps needed to use this software.

Moreover, in order to use this SSM tool in clinical practice, future perspectives entail incorporation of the current programming code into existing segmentation software or to develop a user-friendly interface. In that way, each clinical expert can obtain a reconstruction for a patient with an acetabular fracture for pre-operative treatment planning.

This study has some strengths and limitations. Our SSM describes anatomical variation of a large cohort (*n* = 200), thereby capturing important anatomical features for reconstruction purposes. Moreover, we evaluated real and clinical cases, including patients with elementary and associate type fractures.

The most important limitation of the study is that we assumed symmetry between the left and right hemipelvis. Several studies, including research of Li et al. (2021), show that symmetry exists between the left and right acetabular area of the hemipelvis, with a mean RMSE of 0.91 ± 0.16 mm [25–28]. This implies that the actual reconstruction error is probably even slightly smaller than what we reported. The second limitation is related to the pre-processing of the segmentations, since the SSM requires segmentations with a closed surface. If a (closed) reconstructed shape is compared with a contralateral shape that still has cavities or holes (in for instance the thin bony part of the iliac wing) after the registration process, performance metrics such as RMSE will decrease. This dilutes the true reconstruction performance, thereby making it harder to interpret the results. However, for the SSM such a closed surface is not a prerequisite, thereby making it suitable to be used in clinical practice. The third limitation is related to the registration process. The contralateral shape was registered to the mean shape to ensure point-to-point correspondence with the reconstructed shape. Within the registration process the segmentations are smoothed and adjusted to the mean shape, thereby decreasing noisy features of the segmentation. Even though these adjusted shapes do not perfectly correspond with the original shapes, we believe this did not influence the reconstruction performance. For the SSM to be clinically used, the contralateral hemipelvis is not needed. The only reason the contralateral hemipelvis was used in this study, was to evaluate the SSM’s reconstruction performance. The fourth limitation of our study is that we did not specifically evaluate the reconstruction performance of elderly patients with osteoporotic or deformed bones. In our fracture dataset twenty-six patients (31%) were ≥ 60 years, but the exact number of patients with osteoporotic bones is unknown. The median RMSE of the whole hemipelvis of this specific subgroup was 3.1 (2.8–3.7) mm. This reconstruction performance indicates that reconstructions of elderly, with likely lower bone density, can easily be obtained. Therefore, the greatest advantage of our SSM is that it provides both a database of anatomical variation within a general population and that it can be used to reconstruct acetabular fractures in future patients whose contralateral hemipelvis cannot be used for contralateral mirroring.

## Conclusion

Using a statistical shape model allows for accurate virtual reconstructions of elementary and associate type acetabular fractures within a clinically acceptable range, especially within regions important for plate-fitting. SSM-based reconstructions can serve as a valuable tool for pre-operative planning in clinical practice, particularly when a template of the contralateral hemipelvis for contralateral mirroring is unavailable.

## Electronic supplementary material

Below is the link to the electronic supplementary material.

Supplementary Material 1

Supplementary Material 2

## Data Availability

No datasets were generated or analysed during the current study.
