# Supplementary material for: Automatic virtual reconstruction of acetabular fractures using a statistical shape model
Source: Eur J Trauma Emerg Surg. 2024 Aug 27;50(6):2925–36. doi: 10.1007/s00068-024-02615-7 (PMC11666734; doi:10.1007/s00068-024-02615-7)
Supplement: Supplementary file 1 — Supplementary Material 1 [file 68_2024_2615_MOESM1_ESM.pdf]

Online Resource 1 - Description and computation of six parameters in the regions relevant for plate-fitting

Article title

Automatic virtual reconstruction of acetabular fractures using a statistical shape model

Journal name

European Journal of Trauma and Emergency Surgery

Authors

WA van Veldhuizen[1]

R van Noortwijk[1]

AML Meesters[1,2]

K ten Duis[1]

RCL Schuurmann[1,3]

JPPM de Vries[1]

JM Wolterink[4]

FFA IJpma[1]

Affiliations

1. Department of Surgery, University Medical Center Groningen, Groningen, The Netherlands
2. 3D lab, University of Groningen, University Medical Center Groningen, Groningen, The Netherlands
3. Multimodality Medical Imaging Group, Technical Medical Center, University of Twente, Enschede, The Netherlands
4. Department of Applied Mathematics, Technical Medical Center, University of Twente, Enschede, The Netherlands

Corresponding author

Name: Daniëlle (W.A.) van Veldhuizen

Address: Department of Surgery, Division of Vascular and Trauma Surgery, University Medical Centre Groningen, Hanzeplein 1, 9700 RB Groningen, The Netherlands.

Email address: w.a.van.veldhuizen@umcg.nl

In this Online Resource, the description and computation of the six different parameters in the regions relevant for plate-fitting, is described in detail.
To automatically compute these parameters for reconstructed shapes and intact contralateral shapes, the coordinates needed to be defined on the general mean shape and saved. Accordingly, the indices that correspond to these coordinates were located and used to automatically obtain the coordinates for each reconstructed and contralateral shape. In the main manuscript, for each parameter, the absolute value of the reconstructed shape was subtracted from the value of the contralateral shape. For example, the acetabulum diameter of the reconstructed shape was computed to be 50.8 mm and the diameter of the contralateral shape was 51.5 mm, then a difference of 0.7 mm was reported.
Moreover, for all regional coordinates, a root mean square error (RMSE) was computed between the reconstructed shape and the contralateral shape.

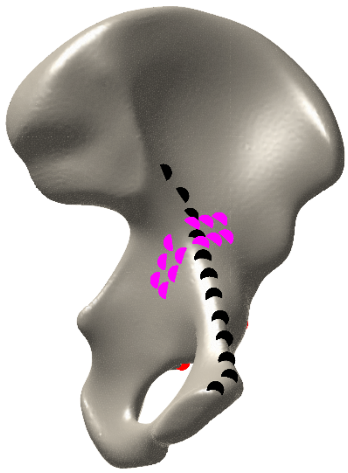

Figure 1. Iliopectineal line coordinates (black dots) and quadrilateral slope (pink dots). The coordinates are projected on the mean shape.

Iliopectineal line length and radius

For the iliopectineal line (black dots in Figure 1), twelve equidistant coordinates were placed along the iliopectineal line. The distance between each successive coordinate was computed, so a total line length in mm could be obtained.
For the iliopectineal curvature, a circle was fitted through three coordinates of the iliopectineal line, namely the first, sixth and last coordinate. This fitted circle has a radius, which was reported for both the reconstructed and contralateral shape (Figure 2). It should be noted that in Figure 2 an example is provided where the difference between the two radii is 12.8 mm. Initially this can be interpreted as a large number, but visually the circles are not that different and clinically it might not be relevant.

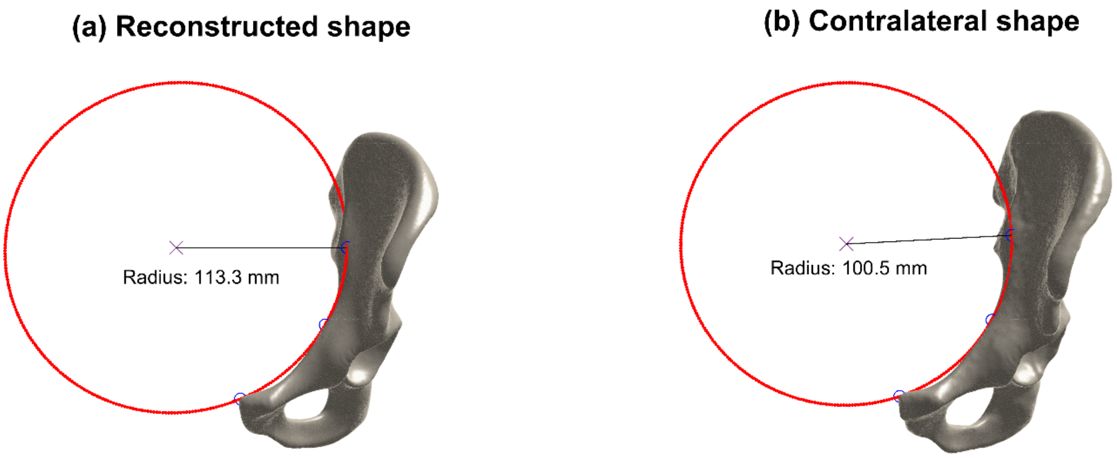

Figure 2. Reconstructed shape **(a)** and contralateral shape **(b)** with fitted circles along the iliopectineal line, providing the radius (red line) and curvature value. The difference between the reconstructed and contralateral shape is 12.8 mm for this case example.

Quadrilateral slope

To obtain the quadrilateral slope, two planes were defined by six coordinates each (pink dots in Figure 1). These two planes were fitted through these six coordinates and the angle between these two planes represents the quadrilateral slope (Figure 3).

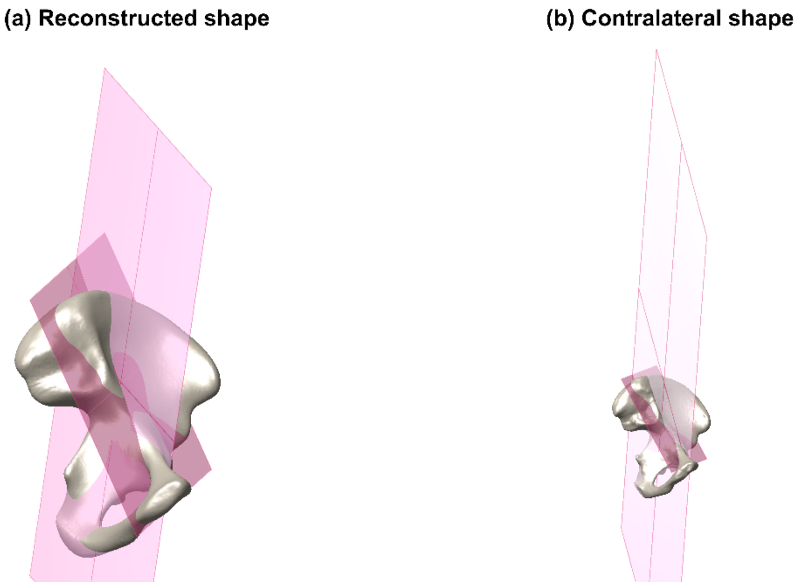

Figure 3. Reconstructed shape **(a)** and contralateral shape **(b)** with two planes plotted in each subplot. The angle between the two planes represents the quadrilateral slope (QLS). The difference between the reconstructed and contralateral shape is 2.8^{o} for this case example.

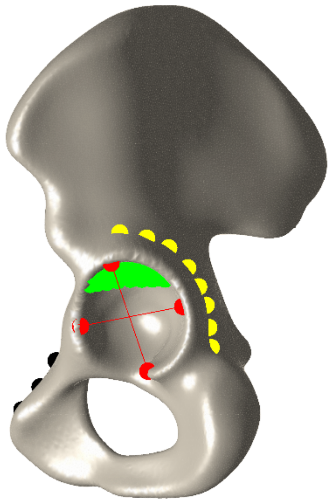

Figure 4. Ischial body line coordinates (yellow dots), acetabulum diameter (red dots) and representation of the weight-bearing acetabulum dome (green dots). The coordinates are projected on the mean shape.

Ischial body line length and radius

For the ischial body line (yellow dots in Figure 4), eight equidistant coordinates were placed along the ischial body. The distance between each successive coordinate was computed, so a total line length in mm could be obtained.

For the ischial body curvature, a circle was fitted through three coordinates of the ischial body line, namely the first, fourth and last coordinate. This fitted circle has a radius, which was reported for both the reconstructed and contralateral shape (Figure 5).

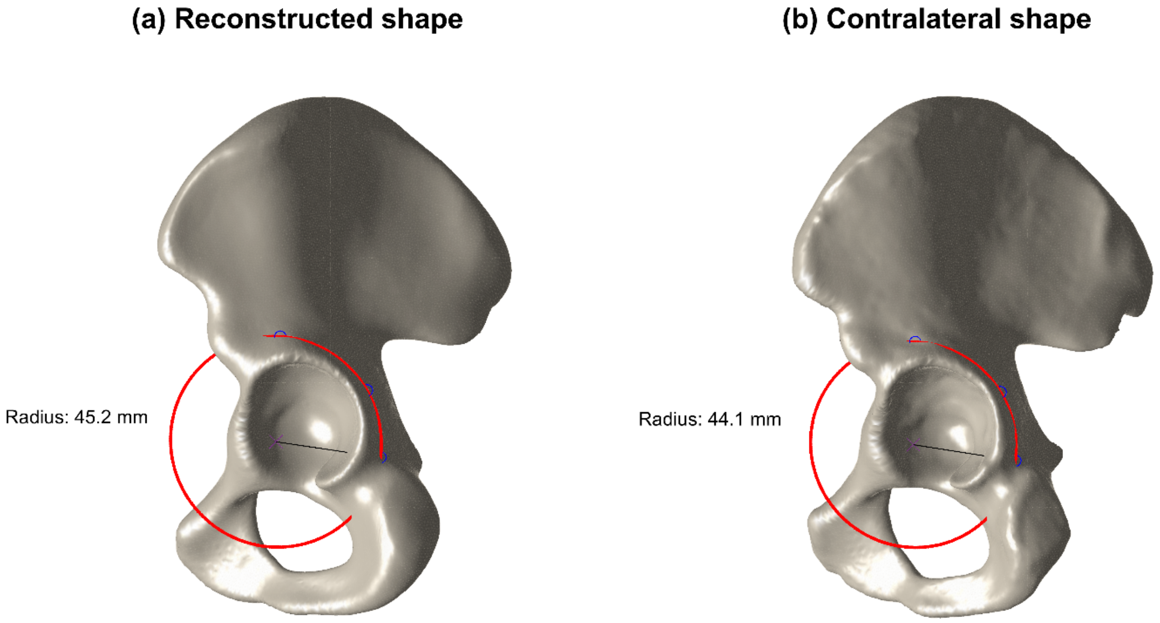

Figure 5. Reconstructed shape **(a)** and contralateral shape **(b)** with fitted circles along the ischial body line, providing the radius (red line) and curvature value. The difference between the reconstructed and contralateral shape is 1.1 mm for this case example.

Acetabulum diameter

The acetabulum diameter was computed by measuring the distance between two opposing coordinates (red dots and lines in Figure 4). The lines of these opposing coordinates are approximately perpendicular to one another. An average distance of the two distances was calculated and reported for both the reconstructed and contralateral shapes.

Weight-bearing acetabulum dome

In total, 30 coordinates were placed in the acetabulum dome to represent the weightbearing part of the acetabulum (green dots in Figure 4).
