# Supplementary material for: Automatic virtual reconstruction of acetabular fractures using a statistical shape model
Source: Eur J Trauma Emerg Surg. 2024 Aug 27;50(6):2925–36. doi: 10.1007/s00068-024-02615-7 (PMC11666734; doi:10.1007/s00068-024-02615-7)
Supplement: Supplementary file 2 — Supplementary Material 2 [file 68_2024_2615_MOESM2_ESM.pdf]

Online Resource 2 – Guide to implement a statistical shape model for reconstruction purposes

Article title

Automatic virtual reconstruction of acetabular fractures using a statistical shape model

Journal name

European Journal of Trauma and Emergency Surgery

Authors

WA van Veldhuizen[1]

R van Noortwijk[1]

AML Meesters[1,2]

K ten Duis[1]

RCL Schuurmann[1,3]

JPPM de Vries[1]

JM Wolterink[4]

FFA IJpma[1]

Affiliations

1. Department of Surgery, University Medical Center Groningen, Groningen, The Netherlands
2. 3D lab, University of Groningen, University Medical Center Groningen, Groningen, The Netherlands
3. Multimodality Medical Imaging Group, Technical Medical Center, University of Twente, Enschede, The Netherlands
4. Department of Applied Mathematics, Technical Medical Center, University of Twente, Enschede, The Netherlands

Corresponding author

Name: Daniëlle (W.A.) van Veldhuizen

Address: Department of Surgery, Division of Vascular and Trauma Surgery, University Medical Centre Groningen, Hanzeplein 1, 9700 RB Groningen, The Netherlands.

Email address: w.a.van.veldhuizen@umcg.nl

In this Online Resource, we provide a guide on how to obtain reconstructions by a statistical shape model (SSM). In our studies, we used Matlab for development of the SSM (MATLAB 2023a, The MathWorks, Inc., MA, USA). Our SSM was based on the adjusted software work by Manu. [1]

Input

A couple of files are needed as input for the SSM to obtain a reconstructed shape:

- A segmentation of the fractured hemipelvis, preferably as a stereolithography (STL) file;
- A segmentation of the mean shape, preferably as an STL file;
- Output of the SSM, as previously developed by our research group, defined by the shape vectors (separate MATLAB file);
- Desired number of principal components (PCs) one wants to take into account. In our case, 15 PCs describe 90% of the total shape variation in our dataset.
- Triangulation matrix of the given (or mean) shape (separate MATLAB file). If the fractured shape was registered, the triangulation matrix of the given and mean shape is the same;
- Optional: a segmentation of the intact contralateral hemipelvis, if available and desired to compute differences between the reconstructed shape and the contralateral shape.

Matlab requirements

Two main steps need to be performed in order to obtain a reconstructed shape, and they both take place in Matlab. The first process involves ensuring anatomical point-to-point correspondence between the fractured shape and the mean shape. We used non-rigid iterative closest point (ICP) algorithm, as was described previously by our study group. [2,3] In the next step, the registered fractured shape will be used as input for the SSM (a so-called function SSMfitter). Next to that, the SSMfitter function requires the file with shape vectors, a mean shape, the triangulation data and the desired number of components.

Output

The output of the SSM consists of the reconstructed shape, which can be saved as an STL file. Moreover, the scores for the principal components (PCs) are also provided. If the intact contralateral hemipelvis was used as input, differences between these two shapes can be computed, such as the root mean square error (RMSE) or regions relevant for plate-fitting can be evaluated, as described in the main manuscript.

References

1 Manu. Shape Model Builder. https://nl.mathworks.com/matlabcentral/fileexchange/49940-shape-model-builder. 05/08/2023.

2 van Veldhuizen WA, van der Wel H, Kuipers HY, Kraeima J, ten Duis K, Wolterink JM, et al. Development of a Statistical Shape Model and Assessment of Anatomical Shape Variations in

the Hemipelvis. *J Clin Med* 2023;**12**(11):3767. Doi: 10.3390/jcm12113767.

3 Manu. nonrigidICP. MATLAB Central File Exchange. https://www.mathworks.com/matlabcentral/fileexchange/41396-nonrigidicp). 08/11/2022.
